# How Each Prosodic Boundary Cue Matters: Evidence from German Infants

**DOI:** 10.3389/fpsyg.2012.00580

**Published:** 2012-12-31

**Authors:** Caroline Wellmann, Julia Holzgrefe, Hubert Truckenbrodt, Isabell Wartenburger, Barbara Höhle

**Affiliations:** ^1^Department of Linguistics, University of PotsdamPotsdam, Germany; ^2^Centre for General Linguistics (ZAS)Berlin, Germany

**Keywords:** infants, language acquisition, speech perception, prosodic bootstrapping, prosodic boundary cues, cue weighting, intonation phrase boundary, headturn preference procedure

## Abstract

Previous studies have revealed that infants aged 6–10 months are able to use the acoustic correlates of major prosodic boundaries, that is, pitch change, preboundary lengthening, and pause, for the segmentation of the continuous speech signal. Moreover, investigations with American-English- and Dutch-learning infants suggest that processing prosodic boundary markings involves a weighting of these cues. This weighting seems to develop with increasing exposure to the native language and to underlie crosslinguistic variation. In the following, we report the results of four experiments using the headturn preference procedure to explore the perception of prosodic boundary cues in German infants. We presented 8-month-old infants with a sequence of names in two different prosodic groupings, with or without boundary markers. Infants discriminated both sequences when the boundary was marked by all three cues (Experiment 1) and when it was marked by a pitch change and preboundary lengthening in combination (Experiment 2). The presence of a pitch change (Experiment 3) or preboundary lengthening (Experiment 4) as single cues did not lead to a successful discrimination. Our results indicate that pause is not a necessary cue for German infants. Pitch change and preboundary lengthening in combination, but not as single cues, are sufficient. Hence, by 8 months infants only rely on a convergence of boundary markers. Comparisons with adults’ performance on the same stimulus materials suggest that the pattern observed with the 8-month-olds is already consistent with that of adults. We discuss our findings with respect to crosslinguistic variation and the development of a language-specific prosodic cue weighting.

## Introduction

The system underlying the prosodic organization of language constitutes a complex linguistic subsystem with strong interfaces to other linguistic domains like the lexicon or the syntax. This paper deals with the correlation between prosodic phrasing and the syntactic structure of utterances which has already been the subject of numerous studies in the area of adult sentence processing as well as of infant language acquisition (e.g., Streeter, 1978; Scott, 1982; Hirsh-Pasek et al., 1987; Sanderman and Collier, 1997; Nazzi et al., 2000; Soderstrom et al., 2003; Peters, 2005). The question unifying these diverse areas of research is whether prosody provides information that can enter into the processing of the syntactic structure of utterances. In language acquisition research this approach is known as the prosodic bootstrapping account (Gleitman and Wanner, 1982), which assumes that infants can exploit acoustic information from their speech input to find solutions for several tasks they are faced with when accessing the grammatical system of their language. In this paper, we will have a closer look at German infants’ sensitivity to the acoustic cues that mark a major prosodic boundary, that is, the intonation phrase boundary (IPB).

There are two properties that render IPBs especially useful within the prosody-syntax mapping. First, a rather clear-cut set of acoustic cues, namely pitch changes, lengthening of preboundary segments, and pauses, is associated with IPBs across different languages (e.g., Vaissière, 1983; Nespor and Vogel, 1986; Price et al., 1991; Wightman et al., 1992; Venditti et al., 1996; Hirst and Di Cristo, 1998; Peters et al., 2005; Féry et al., 2011). Secondly, again crosslinguistically, there exists a high coincidence of IPBs with major syntactic boundaries like sentence and clause boundaries (e.g., Cooper and Paccia-Cooper, 1980; Venditti et al., 1996; Vaissière and Michaud, 2006). Hence, sensitivity to the relevant acoustic cues would provide infants with a strong mechanism for chunking incoming speech into syntactically relevant units without requiring lexical or syntactic knowledge.

Indeed, numerous studies within the prosodic bootstrapping account have demonstrated that infants are equipped with a high sensitivity to prosodic information such as stress, rhythm, and intonation (for an overview, see Jusczyk, 1997). This also holds for the perception of acoustic information that is related to the marking of prosodic boundaries. Research in this area started with some landmark studies that tested infants’ reactions to the presentation of natural speech in contrast to manipulated speech material in which pauses had been inserted at non-boundary positions (Hirsh-Pasek et al., 1987; Kemler Nelson et al., 1989). These studies – using the headturn preference procedure (HPP) – showed that American infants as young as 7–10 months prefer to listen to speech material showing a coincidence of the typical acoustic cues occurring at clausal boundaries compared to materials in which the coincidence of pauses with other prosodic cues had been disrupted. The fact that the same preference occurred with low-pass-filtered material strongly suggests that it is the disturbance of the prosodic organization of the utterances that causes the successful discrimination of both kinds of material. Studies with other languages using the same technique of pause insertion have provided evidence that this discrimination ability is not unique to English-learning infants: German as well as Japanese infants have been found to discriminate speech with pauses at clausal boundaries from speech with pauses inserted at non-boundary positions in their language (Hayashi and Mazuka, 2002; Schmitz, 2008).

Also using pause insertion, Jusczyk et al. (1992) investigated infants’ sensitivity to boundaries of smaller units, namely clause-internal phrase boundaries. In their material, pauses were inserted either before the main verb, that is, at the boundary between the subject and the verb phrase, or after the main verb, that is, within the verb phrase. English-learning 9-month-olds preferred to listen to the materials in which the pause occurred at the phrasal boundary.

Gerken et al. (1994) compared sentences with lexical subjects (e.g., *The caterpillar ate* …) and sentences with pronominal subjects (e.g., *He ate* …) in which pauses had been inserted after either the subject or the verb. As lexical subjects form their own phonological phrase, there is a prosodic boundary between the subject and verb in the corresponding sentences while there is typically no prosodic boundary after a pronominal subject. Only in the lexical subject condition did 9-month-old infants prefer to listen to sentences with pauses after the subjects (e.g., *The caterpillar # ate* …*, He # ate* …) over those with pauses after verbs (e.g., *The caterpillar ate #* …*, He ate #* …). These results again suggest that the prosodic organization – and not the syntactic one – is relevant for infants’ preference for natural material. Taken together, these studies provide evidence that by 9 months infants are sensitive to the acoustic markers at clausal as well as at phrasal boundaries.

More recent work has gone beyond the question of the perception of the acoustic correlates of major boundaries to the question as to whether the occurrence of prosodic boundaries affects the segmentation of continuous speech. Nazzi et al. (2000) were the first to test English-learning 6-month-olds’ use of prosodic boundary cues to segment continuous speech. At the beginning of the experiment infants were familiarized with a sequence of words, once as a prosodically “well-formed” clause (e.g., *Leafy vegetables taste so good*.) and once as a prosodically “ill-formed,” that is, non-clausal sequence that contained an internal clause boundary (e.g., …*leafy vegetables. Taste so good*…). These word sequences had been extracted from two different continuous passages. After familiarization infants were presented with two passages. One of them contained the familiarized prosodically well-formed sequence, the other the prosodically ill-formed sequence, which was now the end and the beginning of two adjacent sentences. This non-clausal unit contained a prosodic boundary that was marked by a pitch change, preboundary lengthening, and a pause. Infants listened significantly longer to the passage containing the clausal sequence than to the passage with the non-clausal sequence. These results suggest that word sequences that constitute a prosodic unit are better recognized than word sequences that span a prosodic boundary. Hence, prosodic boundary cues support the segmentation of clauses within a passage of sentences. These findings were replicated by Soderstrom et al. (2005) with a similar design, but more complex experimental materials. Specifically, it was demonstrated that prosodic boundary cues support English-learning infants’ detection of familiar word sequences even across different passages of fluent speech.

Moreover, with a similar experimental design, Soderstrom et al. (2003) provided evidence that 6-month-old English-learning infants also use prosodic markers to detect syntactic units that are smaller than the clause, namely phrasal units such as noun and verb phrases. Interestingly, phrase boundaries were characterized by preboundary lengthening and pitch cues while there was no perceivable pause at the crucial position. This suggests that for the detection of phrase boundaries pause is not a necessary cue for 6-month-old English-learning infants.

The studies presented so far point to a crucial role of prosodic boundary information in infants’ speech segmentation, especially during the first year of life. However, in a critical analysis of the prosodic bootstrapping account, Fernald and McRoberts (1996) doubt the reliability of acoustic correlates of prosodic boundaries as cues to syntactic units. The authors claim that none of the three markers is a reliable cue to syntactic boundaries as each cue also has non-linguistic functions (e.g., pitch changes for the regulation of affect) or linguistic functions other than syntax (e.g., vowel length as phonemic contrast). This would cause ambiguity of the acoustic correlates of boundaries whenever they occur at non-boundary positions. Fernald and McRoberts’ argument may be weakened if a comprehensive analysis of a corpus of German adult-directed speech conducted by Peters et al. (2005) is taken into account. They found that IPBs were most frequently marked by pitch changes, followed by preboundary lengthening, while the occurrence of pause is rather rare. In addition, the analysis showed that each cue may occur individually, but that in a great majority of the cases boundaries are marked by a coalition of all three or two of the relevant cues. This convergence may decrease the ambiguity of prosodic boundary cues provided that the infant only considers a combination of cues to be a boundary marker.

In fact a detailed study by Seidl (2007) that tested the perceptual impact of each of the prosodic cues provided evidence that English-learning 6-month-old infants rely on a combination of cues in their boundary processing. The investigations were based on the materials and the experimental design used by Nazzi et al. (2000). Seidl successively neutralized each acoustic correlate of the prosodic boundaries in the familiarization sequences. Thereby, the acoustic realization of the cue under investigation no longer differed between the two sequences. The question was whether infants, on the basis of the remaining prosodic cues, would still differentiate the clausal and the non-clausal familiarization sequences and recognize the clausal sequence in the passage during testing.

Infants’ detection of the clausal sequence was not disturbed by the neutralization of the pause cue. This indicates that pitch change and preboundary lengthening were sufficient cues for the 6-month-old English-learning infants, whereas the pause was not necessary. Furthermore, preboundary lengthening also proved not to be a necessary cue, because infants still recognized the clausal sequence when preboundary lengthening was neutralized. However, when the pitch cue was neutralized the infants no longer detected the clausal sequence in the passage. Hence, pitch change proved to be a necessary boundary cue for American infants’ clause segmentation. A further experiment investigated whether pitch change as a single cue would suffice, that is, both preboundary lengthening and pause were neutralized. This kind of acoustic manipulation disturbed infants’ detection of the clausal sequence, indicating that a pitch change alone is not sufficient. In conclusion, a combination of pitch change and preboundary lengthening or pitch change and pause was necessary to trigger clause segmentation in 6-month-old English-learning infants. Seidl (2007) argued that by 6 months English-learning infants do not treat prosodic cues equally, but have, at least partially, become attuned to adults’ weighting of prosodic cues in their native language (Streeter, 1978; Scott, 1982; Aasland and Baum, 2003).

Seidl and Cristià (2008) expanded these investigations by testing 4-month-old English-learning infants with the same materials. In contrast to the 6-month-olds, this younger group was successful in clause segmentation only when pitch change, lengthening, and pause in combination signaled the boundary. Neutralization of one of the prosodic cues led to failure in segmentation. Seidl and Cristià (2008) concluded that 4-month-old English-learning infants segment clauses by considering all prosodic boundary cues.

In a following study, Johnson and Seidl (2008) explored whether infants’ weighting of prosodic boundary cues varies across languages. The experimental design of Seidl (2007) was applied with Dutch material to Dutch 6-month-olds. Like the English-learning infants, the Dutch learners segmented the clausal sequence from the text passage. However, when the pause was neutralized in the familiarization sequences Dutch-learning infants failed to segment the clausal sequence from the text passage. Johnson and Seidl (2008) considered two interpretations. One is related to the strength of the prosodic cues. The magnitude of pitch change and preboundary lengthening might not have been salient enough to trigger the clause segmentation. Acoustic analyses of the stimuli had revealed that the saliency of the pitch reset and the pause duration at the clausal boundary differed in the materials used across the two languages. Compared to the English stimuli the pitch reset in the Dutch stimuli was only half the magnitude, whereas the pause was more than twice as long. However, the qualitative difference in the prosodic cues in the Dutch versus English stimuli might reflect language-specific boundary markings as Dutch compared to English generally tends to have a smaller pitch range (Collins and Mees, 1981; Willems, 1982). Therefore, Johnson and Seidl argued for a different interpretation: by 6 months, with increasing exposure to the native language, Dutch-learning and English-learning infants have developed a language-specific prosodic cue weighting that influences infants’ clause segmentation procedures.

Taken together, these findings indicate that infants’ sensitivity to acoustic cues as prosodic boundary markers is subject to a developmental change during early infancy – perhaps a change from a more general perceptually driven mechanism that relies on a broad set of acoustic cues to a mechanism that is attuned to the specific properties of the target language.

To further investigate the question of an early weighting of prosodic boundary cues, the present study set out to test infants learning German, a language in which we have – at least for adult-directed spontaneous speech – specific knowledge about the frequency of occurrence of prosodic cues at IPBs (Peters et al., 2005), the prosodic unit under investigation in this study. Moreover, from a study with German listeners, findings on adults’ weighting of the relevant acoustic cues are available: in a prosodic judgment task Holzgrefe et al. (2012) tested whether the presence of the cues pitch change and preboundary lengthening in the absence of the pause cue would suffice to signal a boundary. Listeners were presented with coordinated sequences of three names in different prosodic groupings. Their task was to judge the heard sequence as to whether or not it had an internal boundary. The German adult listeners identified the internal boundary when both, a pitch change as well as preboundary lengthening, but no pause, were present in the sequence; however, pitch change alone or lengthening alone was not sufficient. In the present study the same linguistic materials were used to test whether German infants’ processing of prosodic boundary cues is similar to that shown for German adults.

Hence, in contrast to previous studies, we did not present complex clauses (Nazzi et al., 2000; Seidl, 2007; Johnson and Seidl, 2008; Seidl and Cristià, 2008), but well-formed sequences that allowed for a precise acoustic characterization of the phonetic instantiation of the crucial prosodic boundaries which we considered to be the basis for a controlled acoustic manipulation of the stimuli. Thus, going beyond the previous studies with English- and Dutch-learning infants, the results of the infants tested in the current study could be related to findings from adults, allowing a direct comparison of German adults’ and infants’ cue weighting.

Again in contrast to previous studies, we did not test infants’ segmentation, but their discrimination ability. We suggest that infants’ attunement to specific properties of their native language is not only displayed in segmentation tasks as revealed by the work of Johnson and Seidl (2008), Seidl (2007), and Seidl and Cristià (2008). Instead, perceptual reorganization with respect to cue weighting should also be reflected in discrimination performance as has been shown for tone and phonemic contrasts in previous research (Werker and Tees, 1984; Polka and Werker, 1994; Mattock and Burnham, 2006; Mattock et al., 2008). If prosodic boundary cues are perceptually weighted individually, we assume that the less weighted information will contribute less to both discrimination and segmentation.

Experiment 1 served as a baseline to ensure that in our experimental design German-learning infants perceive a boundary signaled by all three prosodic cues. In Experiment 2 we investigated whether the specific combination of a pitch change and preboundary lengthening is sufficient for boundary detection. Hereby, the question whether pause is a necessary cue would be examined. We did not test a combination of two cues that included the pause cue, because we expected that 8-month-olds would discriminate between stimuli with and without a pause easily given that the pause is a rather strong acoustic cue, especially in a mere discrimination task. In fact, in a similar study with younger German infants (Wellmann et al., in preparation) we found that even 6-month-olds are able to use the pause cue. More precisely, a pitch change together with preboundary lengthening was not sufficient for 6-month-olds, but the combination of pause and lengthening was. Thus, a pause, but not a pitch change was a necessary cue for 6-month-olds. This finding moreover suggests that successful boundary detection depends on the specific cue constellation, rather than on the number of boundary cues provided.

After testing the combination of pitch change and preboundary lengthening, we examined the impact of each of the two as single cues: Experiment 3 tested pitch change and Experiment 4 preboundary lengthening.

## Experiment 1: A Baseline Study on Infants’ Sensitivity to Pitch Change, Preboundary Lengthening, and Pause

In Experiment 1, we sought to ensure that 8-month-old German-learning infants are able to perceive a prosodic boundary that is signaled by the three main prosodic cues pitch change, preboundary lengthening, and pause. This would provide a verification of the experimental design and material as suitable for studying the perception of single prosodic boundary cues. As previous research has revealed that infants are sensitive to prosodic boundary information (e.g., Hirsh-Pasek et al., 1987; Nazzi et al., 2000), infants tested in Experiment 1 should be able to perceive a prosodic boundary. Experiment 1 aimed at creating a baseline for the subsequent experiments, in which the constellation of prosodic cues would be systematically varied.

### Materials and methods

#### Participants

Twenty-four 8-month-old infants (12 girls) were tested. The mean age was 8 months, 16 days (range: 8 months, 3 days–8 months, 30 days). All infants who participated in this and the following experiments were from monolingual German-speaking families, born full-term and normal-hearing. Eleven additional infants were tested but their data were not included in the analysis for the following reasons: failure to complete the experiment (2), crying or fussiness (3), mean listening times of less than 3 s per condition (3), technical problems (2), and experimenter error (1).

#### Stimuli

The stimuli consisted of a sequence of three German names that were coordinated by *und* (“and”). The advantage of using coordinated structures instead of clauses lies in the better control of phonological and consequently prosodic parameters. Thus, we used the following three names, which only contained sonorant sounds: *Moni, Lilli, Manu*. This allowed for a reliable measurement of the fundamental frequency – the acoustic correlate of the pitch contour.

Several recordings of the same sequences of names were made in an anechoic chamber equipped with an AT4033a audio-technical studio microphone, using a C-Media Wave soundcard at a sampling rate of 22050 Hz with 16 bit resolution. A young female German native speaker from the Brandenburg area was instructed to read the sequence in two different prosodic groupings, as indicated by different bracketing as in (1).

(1)
  a. (Moni und Lilli und Manu)
  b. (Moni und Lilli) (und Manu)

Each name is a syntactic XP and is correspondingly set off by a phonological phrase boundary from the other names (Gussenhoven, 1992; Truckenbrodt, 1999, 2007). Both sequences contain the same string and are disambiguated either by grouping all three names together as shown in (1a) or by grouping the first two names together and the final one apart as shown in (1b). This disambiguation employs the next higher level of the prosodic hierarchy, that is, the intonation phrase (IP). Thus, sequences of type (1a) are produced as a single IP, that is, without an internal boundary. In contrast, sequences of type (1b) are produced with an IPB after the second name, and consequently consist of two IPs. For each type of prosodic phrasing, the speaker produced six different acoustic realizations (tokens). The intended prosodic grouping was confirmed by two independent listeners who were naïve to the given bracketing.

**Figure 1:**
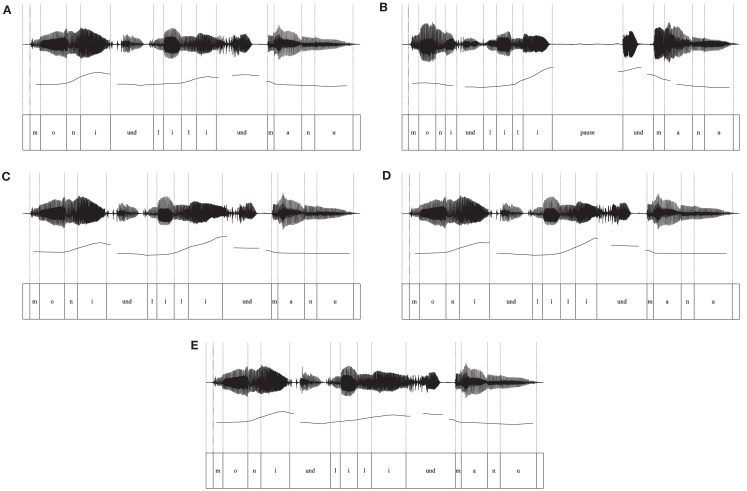
**Oscillograms and pitch contours aligned with the text**. **(A)** Sequence without an internal IPB used in Exp. 1, **(B)** Sequence with an internal IPB used in Exp. 1, **(C)** Sequence with pitch change and preboundary lengthening used in Exp. 2, **(D)** Sequence with pitch change used in Exp. 3, **(E)** Sequence with preboundary lengthening used in Exp. 4.

The presence of the characteristics of an IPB in the sequences of names were confirmed by a detailed acoustic analysis of the recordings using PRAAT software (Boersma and Weenink, 2011). Measurements were carried out at the critical boundary position, namely on and after the second name. The analysis concentrated on the three acoustic correlates of prosodic boundary cues – fundamental frequency (F_0_), the duration of the final vowel, and the pause. Examples of the oscillogram and the fundamental frequency aligned with the segments for sequences without an IPB are shown in Figure 1A, and for sequences with an IPB in Figure 1B. Details of the acoustic analysis are presented in Table 1.

**Table 1:** **Mean values and range of the acoustic correlates of prosodic boundary cues in the experimental stimuli**.

Acoustic correlate	Without an internal IPB	With an internal IPB
	*[Moni und **Lilli** und Manu]*	*[Moni und **Lilli**] [und Manu]*
Pitch rise in Hz	88 (77–110)	220 (197–240)
Pitch rise in semitones	6.7 (5.8–8.2)	14.0 (12.8–14.6)
Maximum pitch in Hz	277 (264–293)	397 (371–422)

	*[Moni und Lill **i** und Manu]*	*[Moni und Lill **i**] [und Manu]*
Final vowel duration in ms	99 (91–110)	175 (162–186)

	*[Moni und Lilli **#** und Manu]*	*[Moni und Lilli]**#** [und Manu]*
Pause duration in ms	0	506 (452–556)

The target word for the analysis was decomposed into four intervals corresponding to the phonetic segments, that is, the single consonantal and vocalic parts of the signal. F_0_ was measured at the midpoint of the first segment and at the position of the maximum F_0_ on the final vowel. The difference between these values was used to calculate the pitch change preceding the boundary. In sequences with an IPB, a pitch rise occurred, starting at the second syllable of the word and leading to a high boundary tone at the final vowel. This pitch change was 2.5 times greater in sequences with an IPB compared to sequences without an IPB (see Table 1 and Figure 1A vs. 1B). A mean pitch reset of 25 Hz from the high boundary tone to the midpoint of the following conjunction *und* (“and”) was measured in sequences with an IPB, whereas the pitch change was only 3 Hz at the same location in sequences without an IPB. Thus, the pitch reset was greater in sequences with a boundary, but compared to Seidl’s (2007) stimuli the overall extent of the reset was rather small, as the conjunction *und* was also uttered on a high pitch level (see the pitch contour in Figure 1B). First and foremost, in our stimuli the pitch cue in sequences with an IPB was provided by the pitch rise on the target name.

Preboundary lengthening was calculated by measuring the length of the final vowel in both prosodic types. Transitions between the final vowel and the onset of the conjunction *und* were not included. The vowel duration was about 1.8 times longer before a boundary compared to the same vowel in the sequence without an internal IPB.

The duration of the pause after the target name had a mean of 506 ms in sequences with an internal IPB. In contrast, no pause was present at this position in sequences without an internal IPB.

To summarize, sequences with an internal IPB clearly revealed the acoustic correlates of the three main prosodic boundary cues similar to IPBs in German spontaneous speech (Peters et al., 2005). A pitch rise occurred on the target name followed by a pitch reset after a pause. Preboundary lengthening was observed at the final vowel of the target name.

Following the acoustic analyses the different recordings (tokens) were used to create sound files for presentation as trials during the experiment. For each prosodic type, the six tokens were randomly concatenated with a silent interval of 1 s inserted between them. In this way, six sound files per prosodic grouping were created such that each file consisted of a different order of tokens. The average duration of tokens without an IPB was 1.76 s (range: 1.71–1.87 s), while it was 2.16 s (range: 2.13–2.2 s) for tokens with an IPB.

To match the sound files of the two prosodic types with respect to length the number of tokens within each file was varied. Files of the grouping with an IPB contained six tokens and had an average duration of 18.97 s. However, files of the condition without an IPB contained seven tokens (i.e., one random token was repeated), leading to an average duration of 19.32 s (range: 19.16–19.43 s).

#### Procedure

The HPP including a familiarization phase (Hirsh-Pasek et al., 1987; Jusczyk and Aslin, 1995) was used in this and all subsequent experiments. During the experimental session, the infant was seated on the lap of a caregiver in the center of a test booth. The caregiver listened to music over headphones to prevent influences on the infant’s behavior. Furthermore, she was instructed not to interfere with the infant’s behavior during the experiment. The experimenter sat in an adjacent room, where she observed the infant’s behavior on a mute video monitor and controlled the presentation of the visual and the acoustic signals by a button box.

Three lamps were fixed inside the booth: a green one on the center wall, and red ones on each of the side walls. Directly above the green lamp on the center wall was an opening for the lens of a video camera. Behind each of the red lights a JBL Control One loudspeaker was mounted. Each experimental trial started with the blinking of the green center lamp. When the infant oriented to the green lamp, it was turned off and one of the red lamps on a side wall started to blink. When the infant turned her head toward the red lamp, the speech stimulus was started, delivered via a Sony TA-F261R audio amplifier to the loudspeaker at the same side. The trial ended when the infant turned her head away for more than 2 s, or when the end of the speech file was reached. If the infant turned away for less than 2 s, the presentation of the speech file continued but the time spent looking away was not included in the total listening time. The whole session was digitally videotaped. The experimenter’s coding was recorded and served for the calculation of the duration of the infant’s headturns during the experimental trials (for comparable experimental setups, see Höhle et al., 2006; Höhle et al., 2009).

Half of the infants were familiarized to the sequences without an IPB (Group 1), while the other half were familiarized to the sequences including an IPB (Group 2). The familiarization was set such that at least 20 tokens in each familiarization condition were presented, that is, when familiarized to sequences without an IPB the familiarization lasted until the infant had accumulated 55 s of listening time. For the familiarization with an IPB the criterion was 63 s of accumulated listening time. This requirement was chosen to match the familiarization duration used in Nazzi et al. (2000).

Two different kinds of familiarization were chosen to control for a possible effect of the prosodic structure of the sequences presented. One might hypothesize that a familiarization to sequences without an internal IPB might be more effective. This is supported by Nazzi et al.’s (2000) findings that infants recognize word sequences that constitute a prosodic unit better than sequences that are a non-unit like our sequences with an IPB. Therefore, we planned to compare the data of both familiarization groups.

The familiarization was followed by a test phase that comprised 12 test trials. In six trials, the sound files without an internal IPB were presented, in the other six trials the sound files of the sequences with an IPB. Thus, half of the test trials contained exactly the same sound files that the infants had previously heard during familiarization, whereas the other half consisted of sound files with the type of prosodic grouping that had not been presented during familiarization. The test trials were grouped in three blocks of four trials each (two with and two without an internal IPB in a random order). Additionally, within each block the side of presentation of the sequences of the two prosodic types was counterbalanced so that the prosodic condition and the side of presentation were not associated. The duration of each experimental session depended on the infant’s behavior and varied between 4 and 6 min.

### Results and discussion

Mean listening times to the test trials with and without an IPB were calculated for each infant. Because all listening times were shorter than 18.97 s (the maximum trial length in the condition with an IPB), an adjustment of the listening times to the longer duration of the trials without an IPB was not necessary.

**Figure 2:**
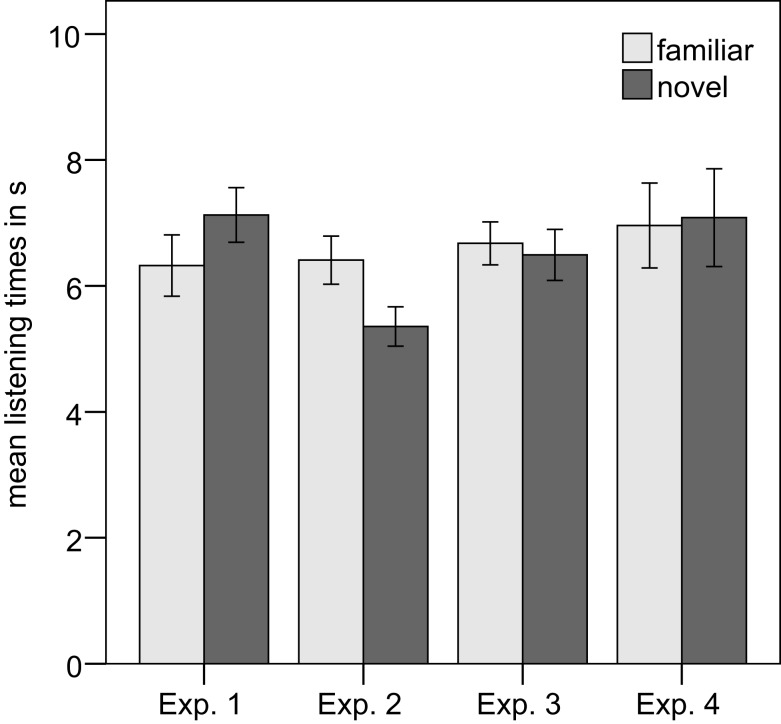
**Mean listening times for Experiment 1–4**. Error bars indicate ±1 SE.

On average, infants listened for 6.32 s (SD = 2.39) to the familiarized prosodic grouping, and for 7.13 s (SD = 2.12) to the novel prosodic grouping (see Figure 2). This difference was significant, *t*(23) = 2.30, *p* = 0.031, two-tailed. Eighteen out of 24 infants had longer listening times to the novel test items. A repeated-measures ANOVA with the within-subject factor familiarity (familiarized versus new prosodic pattern) and the between-subject factor prosodic type (familiarization with versus without an internal IPB) showed a main effect of familiarity, *F*(1,22) = 5.36, *p* = 0.030, and a main effect of prosodic type, *F*(1,22) = 4.44, *p* = 0.047, but no significant interaction between prosodic type and familiarity, *F*(1,22) = 1.237, *p* = 0.278.

**Figure 3:**
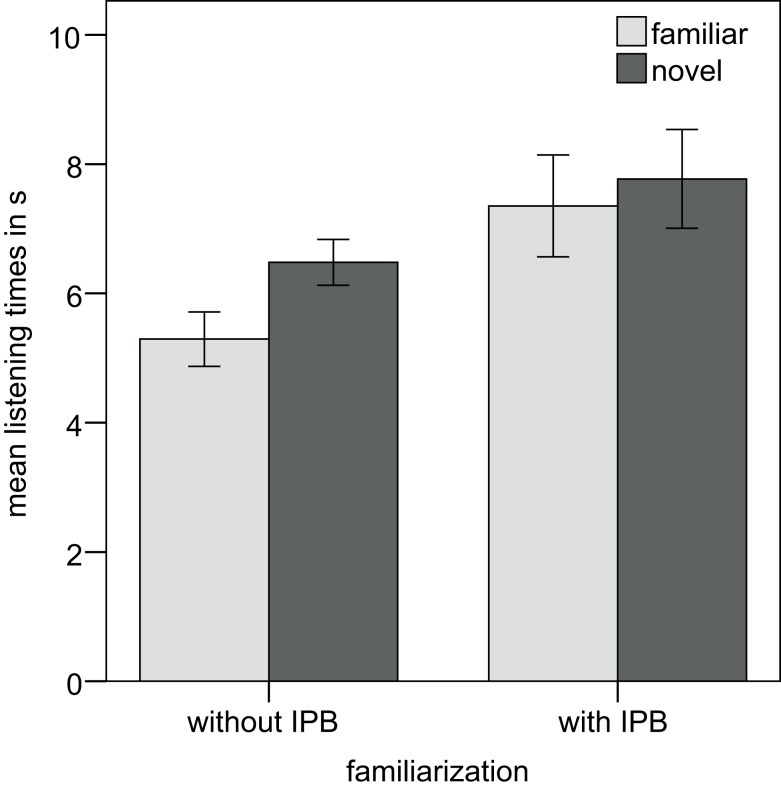
**Mean listening times for Experiment 1, separated by familiarization group**.

A further analysis of the data separated by prosodic type heard during familiarization was conducted. This analysis revealed a significant preference for novel test items in the group familiarized with the sequences without an IPB, *t*(11) = 2.40, *p* = 0.035. The mean listening time to the novel prosodic pattern was 6.48 s (SD = 1.23) and to the familiarized prosodic pattern 5.29 s (SD = 1.46). No such preference was present in the group familiarized with sequences including an IPB, *t*(11) = 0.860, *p* = 0.408. Infants in this group listened to the novel test trials on average for 7.77 s (SD = 2.64) and to the familiar test trials for 7.36 s (SD = 2.73). Mean listening times separated by familiarization group are depicted in Figure 3.

Experiment 1 served as a baseline study to ensure that the stimuli – sequences of names that have two different prosodic groupings – and our experimental design are suitable for studying the perception of prosodic boundary cues in German-learning infants. After being familiarized with one of the two prosodic phrasings, 8-month-old infants showed an overall preference for the novel prosodic grouping. Thus, German-learning infants are able to discriminate the two prosodic groupings. Even though we found no significant interaction between prosodic type and familiarity, a separate analysis of the two familiarization groups revealed that the difference in listening times was significant only when the familiarization strings did not have an internal IPB. Thus, discrimination of sequences with versus without an IPB was affected by the prosodic type heard during familiarization. How can we explain this effect? During familiarization the infants’ task is to build up a representation of the auditory stimulus, to which they will compare the test stimuli. Presumably, infants can more easily build up representations of sequences without an IPB because these are easier to process and memorize, as Nazzi et al.’s (2000) study demonstrated. Secondly, both familiarization conditions differ in the number of IPs: stimuli played to Group 1 do not contain any prosodic boundary cue and, hence, only consist of a single IP, that is, one prosodic unit. In contrast, stimuli presented to Group 2 were sequences including an IPB, which splits the sequences into two separate IPs, that is, two prosodic units.

A study by Mandel et al. (1994) suggests that infants at the age of 2 months already perceive prosodic units as an organizational unit in the speech stream. Infants detected phonetic changes in word sequences when the words were prosodically grouped into a major linguistic unit, but not when the words were presented as isolated words in a list or as a fragment of two adjoining clauses. Mandel et al. argued that the organization of words in a prosodic unit helps infants to process and memorize the speech signal. For our experiment this implies that the representation of the familiarization sequence is built up more easily when the sequence consists of a single prosodic unit, like our sequences without an internal IPB. These are – compared to the sequences with an IPB – easier to process during the familiarization phase and thus can be better remembered during the test phase.

The difference found in the two familiarization groups motivated a modification of the experimental design implemented in the subsequent experiments. As a full design with two separate familiarization conditions was not relevant to our research question, we decided to only use strings without an internal IPB as familiarization stimuli. In doing so, we chose the condition that yielded the most robust results.

In sum, Experiment 1 showed that 8-month-old German-learning infants are sensitive to the presence of an IPB in short coordinated sequences of names when the IPB is marked by the acoustic correlates of the main prosodic boundary cues pitch change, preboundary lengthening, and pause. Hence, not only clauses – like those that were used in previous studies (e.g., Hirsh-Pasek et al., 1987; Nazzi et al., 2000; Seidl, 2007; Schmitz, 2008) – are suitable for investigating infants’ sensitivity to prosodic boundaries. Rather, coordinate structures, which can be carefully controlled for phonological parameters, may serve as stimuli to characterize the impact of each prosodic boundary cue in a discrimination task.

The subsequent experiments contain only one kind of familiarization, namely the familiarization to sequences without an IPB. In these experiments, the number of prosodic boundary cues in the stimuli is reduced stepwise. This is done to determine whether infants’ discrimination ability remains or is disturbed when different constellations of prosodic boundary cues are given.

## Experiment 2: Sensitivity to Pitch Change and Preboundary Lengthening

In Experiment 2, we investigated infants’ sensitivity to two of three prosodic boundary cues, namely pitch change in combination with preboundary lengthening. Specifically, we asked whether the pitch change and the lengthening of the preboundary vowel suffice as boundary cues or whether the pause is a necessary prosodic boundary cue. If pause is a necessary cue for the discrimination of two prosodic groupings, infants were not expected to show significantly different listening times to novel versus familiar test items. In contrast, if pitch change and preboundary lengthening are sufficient cues, we expected a significant listening preference.

### Materials and methods

#### Participants

Sixteen 8-month-old infants (eight girls) were tested. The mean age was 8 months, 11 days (range: 8 months, 1 day–9 months, 8 days). Ten additional infants were tested but their data were not included in the analysis for the following reasons: crying or fussiness (6), mean listening times of less than 3 s per condition (2), and noise (2).

#### Stimuli

In Experiment 2, again sequences with and without an internal prosodic boundary cues were presented. The stimuli without any boundary cues were the same as the stimuli used in Experiment 1. The sequences containing a pitch change and preboundary lengthening were construed from the sequences without an IPB by acoustic manipulation – according to the values that had been measured in the sequences with an IPB recorded for Experiment 1. Hereby, we created two types of stimuli that only differed in fundamental frequency and duration at the critical boundary position, that is, on the second name. Apart from that, the sequences of both prosodic types were acoustically identical.

The manipulation was carried out with the PRAAT software. For duration, the final vowel of the target name was lengthened to 180%. This factor was chosen because in Experiment 1 the crucial vowel was on average 1.8 times longer in sequences with an IPB than in sequences without an IPB.

For the manipulation of the pitch contour, first the sequences without an IPB were stylized (two semitones), that is, the number of pitch points was reduced. The reference values of the fundamental frequency were measured on the target name in the sequences with an internal IPB from Experiment 1 – at the midpoints of the four segments [l], [l], [l], and [i]and at the position of the maximum pitch present on the preboundary vowel. Then, pitch points with the mean values at these time points were inserted at the same positions into the sequences without an IPB. We obtained new stimuli for the prosodic type with pitch change and preboundary lengthening. They contained a natural sounding pitch rise of 212 Hz (13.65 semitones) and a preboundary lengthening with a factor of 1.8. The pitch contour and wave form of a sequence with manipulated pitch and lengthening are depicted in Figure 1C.

To avoid comparing natural with acoustically manipulated stimuli we carried out a slight acoustic manipulation of the sequences without an IPB as well: a stylization of the pitch contour (two semitones). After acoustic manipulation, all sequences were resynthesized using the PSOLA function in PRAAT.

Six differently ordered speech files with the same set of tokens in each prosodic condition were created from the acoustically manipulated sequences. The speech files of the condition without an IPB contained seven tokens (i.e., one random token was repeated) and had an average duration of 18.33 s (range: 18.23–18.43 s). The files of the condition with added pitch and lengthening cues also contained seven tokens (again one random token was repeated) and had an average duration of 18.81 s (range: 18.79–19.01 s).

#### Procedure

The procedure was the same as in Experiment 1 with a modification concerning the familiarization phase. Infants in Experiment 2 were only familiarized to sequences without an IPB, but not to sequences with boundary cues. The familiarization lasted until at least 20 sequences had been presented leading to a minimum duration of 52 s.

### Results and discussion

Infants oriented on average for 6.41 s (SD = 1.53) to the familiarized prosodic grouping, and for 5.36 s (SD = 1.25) to the novel prosodic grouping (see Figure 2). This difference was significant, *t*(15) = -3.59, *p* = 0.003, two-tailed. Thirteen of 16 infants had longer listening times to the familiar test items.

Experiment 2 tested whether German-learning infants still perceive an IPB when only a subset of prosodic cues, pitch change, and preboundary lengthening, is present. A significant familiarity effect was displayed indicating that the infants were able to discriminate the stimuli of the two prosodic patterns in Experiment 2. Interestingly, the direction of preference reversed from Experiment 1 to Experiment 2. While infants in Experiment 1 preferred to listen to the novel prosodic pattern, in Experiment 2 the familiar pattern was preferred. According to the model by Hunter and Ames (1988), this shift in preference can be explained by higher task demands in Experiment 2. Hunter and Ames claimed that the direction of preference is affected by three factors: age, duration of familiarization, and task difficulty. As we held the first two factors constant, we assume that the shift in preference from Experiment 1 to Experiment 2 is caused by increased task difficulty: if only two instead of three prosodic cues mark the difference between the stimuli, it becomes harder to distinguish both conditions as less information is available. In turn, the task of discriminating the two prosodic patterns is more difficult and leads infants to a preference for the familiar sequences. Hence, for German 8-month-olds pitch change and preboundary lengthening in combination are sufficient. Pause is not a necessary boundary cue, however, processing different prosodic groupings without the information provided by the pause cue seems to be more demanding.

## Experiment 3: Sensitivity to Pitch Change

Experiment 2 showed that German infants are able to discriminate the two prosodic groupings when a boundary is signaled by a pitch change and preboundary lengthening in combination. In Experiment 3 we asked whether only one cue, the pitch change, is sufficient for German 8-month-olds to perceive a boundary.

### Materials and methods

#### Participants

Seventeen infants (seven girls) were tested. The mean age was 8 months, 13 days (range: 8 months, 4 days–8 months, 29 days). Six additional infants were tested but their data were not included in the analysis for the following reasons: crying or fussiness (4), and mean listening times of less than 3 s per condition (2).

#### Stimuli

In Experiment 3, sequences without an IPB and sequences with an inserted pitch rise were contrasted. For the condition without an IPB the same sequences as in Experiment 2 were used. For the condition with added pitch cue a manipulation of the pitch contour was carried out similar to that in Experiment 2: a pitch rise was inserted on the second name of the six sequences without an IPB. In contrast to the stimuli in Experiment 2 no duration manipulation was conducted. Thus, the pitch change with the high boundary tone was the only signal of an IPB (see Figure 1D). From these pitch-manipulated sequences six differently ordered speech files were created with seven tokens per file (i.e., one of the six exemplars was randomly repeated). The speech files of the condition without an IPB were the same as in Experiment 2. The average duration of the speech files was the same in both prosodic conditions as there was no duration manipulation (*M* = 18.33 s; range: 18.23–18.43 s).

#### Procedure

The procedure was the same as in Experiment 2. Infants were familiarized to sequences without an IPB until at least 20 sequences had been presented. This led to a minimum duration of 52 s.

### Results and discussion

Infants listened on average for 6.68 s (SD = 1.41) to the familiarized prosodic grouping and for 6.49 s (SD = 1.67) to the novel prosodic grouping (see Figure 2). This difference was not significant, *t*(16) = 0.522, *p* = 0.609. Ten of 17 infants had longer listening times to the familiar test items.

In Experiment 3 only a pitch rise indicated a different prosodic grouping. Neither a pause nor lengthening of the preboundary vowel was present. The infants did not differentiate between sequences with added pitch cue and sequences without an IPB. Hence, the presence of a pitch change alone is not sufficient for German infants to perceive a prosodic boundary.

Apart from the specific cue constellation presented, Experiment 3 generally differs from Experiment 2 with regard to the number of IPB cues provided in the stimuli, that is, whereas in Experiment 2 two boundary cues were available, in Experiment 3 we only inserted one cue. Hereby, the boundary is generally less marked in Experiment 3. The failure to discriminate the two conditions could hence be due to the mere number of cues being relevant for boundary detection, instead of the specific kind of cue or cue constellation (but see General Discussion).

## Experiment 4: Sensitivity to Preboundary Lengthening

German 8-month-olds are able to perceive an IPB when a pitch change and preboundary lengthening occur together (Experiment 2) but not when only a pitch change is present (Experiment 3). Experiment 4 tested whether preboundary lengthening as a single boundary cue is sufficient.

### Materials and methods

#### Participants

Sixteen infants (eight girls) were tested. The mean age was 8 months, 10 days (range: 7 months, 30 days–8 months, 29 days). Six additional infants were tested but their data were not included in the analysis for the following reasons: failure to complete the experiment (1), crying or fussiness (2), and mean listening times of less than 3 s per condition (3).

#### Stimuli

In Experiment 4, sequences without an IPB and sequences with inserted preboundary lengthening were contrasted. For the condition without an IPB the same sequences as in Experiment 2 were used. For the condition with inserted preboundary lengthening a manipulation of the duration of the final vowel was carried out similar to that in Experiment 2: in six exemplars of the sequences without an IPB the final vowel was lengthened to 180% (see Figure 1E for an example). The sequences were concatenated in a random order to speech files.

The speech files of the condition without an IPB were the same as in Experiment 2. They contained six different tokens and had an average duration of 18.33 s (range: 18.23–18.43 s). The speech files of the condition with preboundary lengthening also contained six tokens and lasted for 18.89 s on average (range: 18.79–19.01 s).

#### Procedure

The procedure was the same as in Experiment 2. Infants were familiarized to sequences without an IPB until at least 20 sequences had been presented. This led to a minimum duration of 52 s.

### Results and discussion

The listening time in one individual trial of the condition with the lengthening cue exceeded the duration of the longest speech file in the condition without an IPB. Therefore, the listening time in this trial was reduced to the maximum trial length of sequences without an IPB, which was 18.43 s.

The mean listening time to the familiarized prosodic grouping was 6.96 s (SD = 2.7) and to the novel pattern 7.08 s (SD = 3.1; see Figure 2). This difference was not significant, *t*(15) = -0.221, *p* = 0.828. Nine of 16 infants had longer listening times to the familiar test trials.

Experiment 4 suggests that preboundary lengthening as a single cue is not sufficient to trigger the perception of a prosodic boundary in German 8-month-old infants. However, in combination with a pitch cue, as tested in Experiment 2, it becomes an effective boundary marker. As for Experiment 3, we also have to consider that the insufficiency of preboundary lengthening alone compared to its effectiveness in combination with a pitch change could also be explained by the number of cues (but see General Discussion).

## General Discussion

The aim of the present study was to specify the relevance of pitch change and preboundary lengthening as combined and as single prosodic cues in German-learning infants’ perception of major prosodic boundaries. Experiment 1 showed that 8-month-olds are able to discriminate different prosodic groupings – specifically, familiar sequences without a prosodic boundary from unfamiliar sequences with a prosodic boundary – when the boundary is clearly marked by all three boundary markers.

In further experiments stimuli were acoustically manipulated with respect to pitch and preboundary lengthening. We focused on investigating infants’ processing of boundaries in the absence of the pause cue. Pauses are perceptually highly salient and we assumed that in a discrimination task like ours, infants would easily detect the presence of a pause. Especially in short coordinated structures as used in this study pauses are easy to notice as they constitute approximately a fourth of the overall duration of the sequence. Furthermore, we know from other studies (Hirsh-Pasek et al., 1987; Jusczyk et al., 1992; Schmitz, 2008; Wellmann et al., in preparation) that infants by the age of 6–10 months are highly sensitive to pauses.

When we manipulated the stimuli such that only a pitch change and preboundary lengthening indicated the presence of an IPB (Experiment 2), infants still detected the boundary. We concluded that pause is not necessary, but it seems to ease infants’ processing. This was indicated by a shift in preference from a novelty effect in Experiment 1 to a familiarity effect in Experiment 2. We argued that higher task demands in Experiment 2 are responsible for the preference for familiar stimuli (see Hunter and Ames, 1988). In Experiments 3 and 4 the impact of the single prosodic cues pitch change and preboundary lengthening were tested. Sequences with pitch as a single cue (Experiment 3) were not differentiated from sequences without any boundary cue. Nor was preboundary lengthening alone (Experiment 4) sufficient to trigger the perception of a boundary. This might indicate that infants do not take single cues into account, as cue combinations are very frequent whereas the occurrence of single cues is rather rare (Peters et al., 2005). However, the weighting of prosodic boundary cues might depend on the strength of the specific cue, that is, its phonetic magnitude. When implementing the cues in Experiments 2–4 we used the acoustic values measured in natural sequences that contained all three cues. It is conceivable that the specific strength of each cue in production depends on the constellation of cues, that is, when a cue occurs alone or in a subset its magnitude might be larger than when it occurs together with all main cues. Thus, it remains possible that a larger pitch rise in Experiment 3 or longer preboundary lengthening in Experiment 4 might have been sufficient to trigger boundary perception by a single cue. We also considered the reduced number of boundary cues as an explanation for the insufficiency of the single cues compared to their occurrence in combination. However, in a study with 6-month-olds (Wellmann et al., preparation) we found that pause, but not a pitch change, was sufficient though the number of cues was kept constant. Therefore, we argue that the specific cue constellation, and not the number of cues, is decisive for the detection of a boundary.

Another restriction when interpreting the data concerns the fact that the stimuli presented during the test phase differed across experiments in the presence or absence of boundary cues, but potentially also with respect to their naturalness. Thus, infants’ different performance patterns could be due to infants’ disliking of one kind of stimuli in one but not the other experiment. Pitch change or preboundary lengthening might be effective as single cues when produced naturally, but infants could find stimuli with a single inserted cue odd, thus, would not pay attention and consequently fail to discriminate test stimuli. Hereby, infants’ cue weighting and their liking of stimuli might be confounded. However, when editing the stimuli with inserted cues, we took special care to create stimuli that are perceptually distinguishable, but comparably natural sounding in all experiments. Hence, we rather argue that the different performance patterns suggest that perception depends on the specific cue constellation: pitch change and preboundary lengthening in combination are sufficient to trigger boundary perception in German 8-month-old infants and hence, pause is not a necessary cue. Whether pitch change or preboundary lengthening is a necessary cue cannot be answered from these experiments. Still, both of them are not sufficient as single boundary cues: when they occur individually, stimuli are not differentiated from sequences without prosodic boundary marking – at least if the single cues are presented with the same acoustic parameters as when they occur combined.

In summary, two parallels of these findings to previous research are obvious: first, they resemble findings on the processing of these cues in German adults (Holzgrefe et al., 2012), and secondly, they show a strong overlap with the findings by Seidl (2007) for English-learning infants. Both parallels will be discussed separately in the following section.

To our knowledge, the present study is the first that has used the same material with infants that had previously been used with adults in a prosodic judgment task (Holzgrefe et al., 2012). In this study, adults were asked to interpret the aurally presented sequences as having no internal boundary [a and b and c], or as having an internal boundary after the second name [a and b] [and c]. The effects that the specific prosodic cues had on these decisions mirror the pattern we found with the German-learning infants: sequences that provided pitch change or preboundary lengthening as single cues either were judged as having no boundary or listeners performed at chance level. However, when a combination of pitch change and preboundary lengthening occurred in the sequence, they were clearly identified as consisting of two prosodic units. Moreover, infants’ behavior in our study is in line with the distribution of prosodic boundary cues found in spontaneous speech of German adults (Peters et al., 2005): first, the majority of IPBs are marked by a coalition of cues. Secondly, compared to pitch change and preboundary lengthening, pause is a rather rare marker of IPBs. This suggests that pause is not reliable and listeners should be able to cope without it.

It is rather surprising that the experiments with the adults and the infants show exactly the same pattern of results with respect to cue effectiveness even though the tasks that had to be performed by the participants were clearly different: while the adults had to exploit the acoustic information to assign a prosodic phrasing to the utterances, the children only had to discriminate between the different prosodic contours. If we consider these findings in the light of Johnson and Seidl’s (2008) assumption that a language-specific weighting of prosodic boundary cues takes place, our results suggest that the German 8-month-olds have already attuned to the German system as they show a parallel pattern of responding to the cues to that of adults. Furthermore, our results indicate that cue weighting leads to a perceptual reorganization that has an effect on the ability to discriminate verbal materials containing the relevant phonetic information.

Additional empirical support for this conclusion is required and may come from crosslinguistic studies that compare children learning languages that exhibit relevant differences in the acoustic instantiation of prosodic boundary cues. In addition, one may compare the current findings to the performance of younger infants. This would allow a developmental trajectory to be followed from a language-general perceptual system that is not yet fully adapted to the properties of the phonological system of the ambient language to a language-specific perceptual system that is attuned to these properties.

Crosslinguistic research in the area of the processing of prosodic boundaries is still sparse. Additionally, a crosslinguistic comparison may be impeded because of differences in the experimental material of our and previous studies: we used coordinated noun phrases, whereas previous studies on English and Dutch (Seidl, 2007; Johnson and Seidl, 2008) presented clauses. Even though both kinds of material have a different syntactic structure, the prosodic structure is similar. Clause boundaries in Seidl’s (2007) and Johnson and Seidl’s (2008) studies coincide with IPBs. In our sequences of names each name forms a phonological phrase. To convey the intended internal grouping, that is, separating the first two names from the third, our speaker needed to group the first two names into a larger prosodic unit by producing a larger prosodic boundary after the second name. In line with current models of prosodic phrasing (Gussenhoven, 1992; Truckenbrodt, 1999, 2007) we argue that therefore the first two names of the internally grouped sequences constitute an intonation phrase. This account is supported by the acoustic analysis we carried out on the respective IPB cues. Hence, even though the stimuli differ across studies, the prosodic level under investigation is comparable allowing us to compare ours and previous findings crosslinguistically. German infants’ behavior compared to American 6-month-olds’ (Seidl, 2007) shows no indications of crosslinguistic variation. Like the German infants in our study, the 6-month-old American infants did not provide any evidence of detecting a boundary when it was solely cued by pitch change or preboundary lengthening, but only if a combination of these cues occurred in the stimuli. However, given the high overlap in the prosodic systems of English and German, the missing crosslinguistic variation could simply reflect the fact that the two languages do not differ crucially in the area under investigation.

However, a comparison of the results of the experiments with German- and English-learning infants on the one hand and Dutch-learning infants on the other gives some indications of crosslinguistic variation. While the 6-month-old Dutch infants tested by Johnson and Seidl (2008) needed a pause to detect the prosodic boundary, the German and American infants were able to perceive a boundary with pitch change and preboundary lengthening only. This might indicate a true crosslinguistic variation between German and Dutch and English and Dutch.

Regarding the difference observed between the German and Dutch infants’ reliance on the prosodic cues, we have to take into account that it may arise from a purely developmental change. The Dutch infants were 2 months younger than the German ones. It is thus possible that older Dutch babies will be able to detect prosodic boundaries that are not marked by a pause. In addition, it is feasible that German 6-month-olds will not detect a prosodic boundary when no pause is present. This would suggest a developmental change in prosodic cue perception from 6 to 8 months in Dutch and German infants. Future studies comparing German and Dutch infants of the same age will have to disentangle whether the observed difference is due to crosslinguistic variation or is caused by developmental aspects.

Regarding the difference between English- and Dutch-learning infants’ sensitivity to prosodic boundary markers, Johnson and Seidl (2008) took this as an indication of the emergence of a language-specific cue weighting, as the results reflected differences in the way that the prosodic boundaries were marked in the Dutch material and the English material, with a longer pause but smaller pitch reset in Dutch as compared to English. Additional evidence for this view comes from the study by Seidl and Cristià (2008), which revealed that younger, 4-month-old English-learning infants only rely on a combination of all three cues. The authors argued that younger infants’ perception reflects holistic mechanisms that do not depend on language-specific factors. Later in development, infants follow an analytical segmentation strategy that implies language-specific processing (Seidl, 2007). This indicates a developmental shift from 4 to 6 months of age. Based on this reasoning, a further study with German-learning infants younger than the age tested in our study would be necessary to provide more evidence for this kind of developmental change.

Furthermore, it would be highly interesting to look at languages in which the way prosodic boundaries are marked is more different than in the closely related languages English, German, and Dutch. The advantage of the linguistic material used in this study is that it can easily be adapted to other languages. One relevant language to look at would be French. Two features might lead to a greater saliency of preboundary lengthening. First, French does not have lexical stress and thus has no pitch accents. In languages without pitch accents syllable duration is much less varied within phrases. Secondly, French is a syllable-timed language. The inventory of syllable types is smaller in syllable-timed than in stress-timed languages. Smaller syllable inventories comprise simpler syllables, whereas languages with more syllable types tend to have heavier syllables (Ramus et al., 2000). Consequently, syllable duration is less varied in syllable-timed than in stress-timed languages. Both aspects, no lexical stress and a smaller syllable inventory, lead to the assumption that whenever syllables are lengthened, namely phrase-finally, this provides a clear acoustic contrast to phrase-internal syllable durations. Empirical evidence for a greater phonetic extent of preboundary lengthening comes from a production study with German and French adults by Féry et al. (2011). They found that the difference in duration between phrase-internal and phrase-final words was significantly higher in French speakers than in German speakers, who used preboundary lengthening to a smaller degree. Thus, preboundary lengthening might be a more important cue for the perception of prosodic boundaries in French adults and infants compared to the speakers and learners of the languages looked at so far. Again, this question is left open for further research.

Also, tone languages that deploy lexical tones on each syllable should be studied (e.g., Chinese). Where pitch is used to encode lexical distinctions, its role in encoding boundaries is reduced (Fernald and McRoberts, 1996). Therefore, one can hypothesize that infants acquiring such a tone language focus more on other boundary cues, like pause and preboundary lengthening. Pitch would then be perceptually weighted less in this kind of tone language than in non-tone languages.

The results of our study contribute in an important way to our understanding of how prosodic information may support children’s early phrasing of incoming linguistic material and hence provide further evidence for the prosodic bootstrapping account. Fernald and McRoberts (1996) outlined the unreliability of prosodic cues due to their multiple functions. Our results as well as Seidl’s (2007) data show that infants only consider a combination of at least two cues as a marker for a prosodic boundary – and even younger infants rely on the convergence of all cues that serve as prosodic boundary markers (Seidl and Cristià, 2008). With these constraints infants have a powerful mechanism to make specific use of these correlations of cues as boundary markers and to ignore the same acoustic information when it is not accompanied by correlating cues.

## Conflict of Interest Statement

The authors declare that the research was conducted in the absence of any commercial or financial relationships that could be construed as a potential conflict of interest.
